# Modelling the Gut Fungal-Community in TIM-2 with a Microbiota from Healthy Individuals

**DOI:** 10.3390/jof9010104

**Published:** 2023-01-12

**Authors:** Evy Maas, John Penders, Koen Venema

**Affiliations:** 1 Centre for Healthy Eating & Food Innovation, Maastricht University—Campus Venlo, Villa Floraweg 1, 5928 SZ Venlo, The Netherlands; 2 Euregional Microbiome Center, P. Debyelaan 25, 6229 HX Maastricht, The Netherlands; 3 Department of Medical Microbiology, School of Nutrition and Translational Research in Metabolism (NUTRIM) and Care and Public Health Research Institute (Caphri), Maastricht University, P. Debyelaan 25, 6229 HX Maastricht, The Netherlands

**Keywords:** fungi, gut microbiota, in vitro colon-model, ITS2 sequencing, dietary intervention

## Abstract

Most research on the human microbiome focuses on the bacterial component, and this has led to a lack of information about the fungal component (mycobiota) and how this can influence human health, e.g., by modulation through the diet. The validated, dynamic computer-controlled model of the colon (TIM-2) is an in vitro model to study the microbiome and how this is influenced by interventions such as diet. In this study, it was used to the study the gut fungal-community. This was done in combination with next-generation sequencing of the ITS2 region for fungi and 16S rRNA for bacteria. Different dietary interventions (control diet (SIEM), high-carbohydrate, high-protein, glucose as a carbon source) were performed, to see if diet could shape the mycobiome. The mycobiome was investigated after the adaptation period, and throughout the intervention period which lasted 72 h, and samples were taken every 24 h. The fungal community showed low diversity and a greater variability when compared to bacteria. The mycobiome was affected most in the first hours of the adaptation period. Taxonomic classification showed that at the phylum-level *Ascomycota* and *Basidiomycota* dominated, while *Agaricus*, *Aspergillus*, *Candida*, *Penicillum*, *Malassezia*, *Saccharomyces*, *Aureobasidium*, *Mycosphaerella*, *Mucor* and *Clavispora* were the most abundant genera. During the intervention period, it was shown that the change of diet could influence the diversity. Clustering of samples for different time points was analyzed using Bray–Curtis dissimilarities. Samples of t0 clustered together, and samples of all other time points clustered together. The Bray–Curtis-dissimilarity analysis also showed that for the different dietary interventions, samples treated with glucose clustered together and were different from the other groups (*p* < 0.05, PERMANOVA). Taxonomic classification showed that the genera *Alternaria*, *Thanatephorus*, *Candida* and *Dekkera* differentially changed for the various diet groups (*p* < 0.05, Kruskal–Wallis). These results show that the mycobiota could be modelled in TIM-2; however, the low diversity and high variability make studying fungal, as compared to bacterial, communities, much more challenging. Future research should focus on the optimization of the stability of the fungal community to increase the strength of the results.

## 1. Introduction

The human gut harbors a complex microbial community that not only consists of bacteria, but also of fungi and viruses. Most of the microbiome research focuses on the bacterial component, thereby neglecting the fungal community, or mycobiome. Gut fungi make up approximately 0.1% of the gut microbiome [1], but in size they take up a considerable volume of the gut ecosystem. Although there are already some studies that show that the fungal community correlates with diseases and disorders [2,3,4], the exact role of fungi is unknown. In addition, ways to modulate the fungal population in the gut have not been explored extensively. More research in this area can help in understanding the role of gut fungi on (intestinal) health.

The balance of the microbial community can be disturbed by antibiotic use, and the elimination of bacterial species can lead to fungal overgrowth [5]. A well-known example is the opportunistic pathogen *Candida*, where infections with *Candida* can occur after antibiotic treatment [6,7]. Diet has an important influence on the intestinal fungi [8]. Moreover, it is a source of fungi that can pass into or even colonize the gut [9]. There are several examples that show that diet can have an influence on the gut mycobiome; for example, it was shown that mice on a high-fat diet had a different mycobiota compared to mice on a standard diet [10], and that *Methanobrevibacter* and *Candida* abundance was correlated with a high-carbohydrate diet [11].

The use of a predictive in vitro model can provide important insights into the direct impact of diet on the human-gut fungal community. The validated, dynamic computer-controlled model of the colon (TIM-2), has been extensively used to study the bacterial microbiome; for a review see [12]. Different interventions can be carried out, such as dietary, pharmaceutical or nutraceutical (e.g., probiotics) [13,14]. In addition, the microbial inoculum can be chosen to fit the target group, such as children, the elderly, or a specific patient group [15,16,17]. The pooled microbiota used in the model is demonstrated to represent a standardized microbiota and allows the performance of a series of experiments with the same starting microbiota [18]. The colon environment is mimicked by controlling the pH, temperature and low oxygen-levels. The model also makes use of a dialysis system to avoid the accumulation of metabolites, which keeps the metabolite levels within a physiological range. Samples can be taken from the lumen and the dialysate to give insight in the microbiome and metabolites. The model has been validated and extensively used to study the bacterial community, but can also help to study fungi or even the fungal–bacterial interactions. However, prior to the use of TIM-2 to study fungi or fungi–bacteria interaction, proof is required that the model is suitable for maintaining a certain mycobiome from a fecal origin, for which the physiological and nutritional conditions might differ from bacteria.

Historically, fungi were studied with culture-dependent methods, which gives a distorted image of the fungi present in the gut. With the rise of culture-independent techniques, such as sequencing, it is now possible to study fungi in a different way, such that species that are non-culturable under standard culture conditions can also be detected [19]. Most commonly used is the amplicon-based sequencing of the internal-transcribed-spacer unit (ITS) [20]. To start to address the knowledge gap in the role of the fungal community in health and disease, here, ITS sequencing combined with in vitro modelling was used as a tool to gain insight into fungal-community dynamics and fungal–bacterial interactions. Therefore, the aim of this study was to develop and optimize the use of the TIM-2 system, e.g., with respect to media, pH or other conditions, in combination with ITS2 sequencing to mechanistically study the fungal population in the human gut, and study ways to modulate the fungal community.

## 2. Materials and Methods

### 2.1. Test Products

For the adaptation period and as a control diet, simulated ileal efflux medium (SIEM), which simulates the undigested components of a diet that reaches the colon, was used as described previously [21]. SIEM consists of 100 g CHO-medium (containing 12 g/L pectin, 12 g/L xylan, 12 g/L arabinogalactan, 12 g/L amylopectin, 100 g/L starch), 25 g TBCO 6.25× (containing 270 g/L Tween80, 375 g/L bactopepton, 375 g/L casein, 6.25 g/L ox-bile), 2 g MgSO_4_ (50 g/L), 2 g cysteine (20 g/L), 0.2 mL vitamin mixture (containing 1 mg/L menadione, 2 mg/L D-biotin, 0.5 mg/L vitamin B12, 10 mg/L pantothenate, 5 mg/L nicotinamide, 5 mg/L p-aminobenzoic acid and 4 mg/L thiamine), 4 mL salts solution (containing 4.7 g/L K_2_HPO_4_∙3H_2_O, 8.4 g/L NaCl, 0.8 CaCl_2_∙2H_2_O, 0.009 g/L FeSO_4_∙7H_2_O, 0.02 g/L haemin) and 1 mL antifoam emulsion. For the dietary interventions, modifications of SIEM were prepared (Table 1). To create a high-carbohydrate diet (10× CHO), SIEM was modified by diluting ten times the concentration of TBCO, while keeping the rest of the ingredients the same. For the high-protein diet (0.1× CHO), SIEM was modified by the dilution of CHO ten times, where the rest of the ingredients stayed the same. In the diet with glucose as a carbon source, CHO was replaced by 15 g glucose.

### 2.2. TIM-2 Model

To create an anaerobic environment, the TIM-2 model was flushed with N_2_ prior to inoculation and throughout the experiment. The pH and temperature were set to mimic a healthy adult at 5.8 (mimicking the proximal colon) and 37 °C, respectively. The pH was maintained by computer-controlled addition of 2 M NaOH. Accumulation of fermentation products was avoided with the use of a hollow-membrane dialysis system. The growth media (Table 1) were added constantly, at a rate of 2.5 mL/h in the adaptation and test period, leading to the introduction of 45 mL SIEM in the adaptation period and 180 mL of test medium in the intervention period. The volume in the units was kept constant with the use of a level sensor. For the experiments, four TIM-2 units were run at the same time, in parallel. The model is described in more detail previously [12].

### 2.3. Fecal Samples

Fecal samples were collected from healthy adult volunteers (*n* = 6, male:female 50:50) and homogenized under anaerobic conditions to create a standardized pool, as described by Aguirre et al. [18]. All donors provided informed consent. The fecal pool was snap-frozen in liquid nitrogen and stored in −80 °C until use. All experiments were performed with the same fecal pool, and thus the same starting microbiota/mycobiota, which allowed direct comparison between experiments. On the day of the experiment, four tubes were taken out of the freezer and thawed in a water bath for 1 h at 37 °C, after which they were mixed under anaerobic conditions with an equal volume of dialysate (which contained 2.5 g/L K_2_HPO_4_·3H_2_O, 4.5 g/L NaCl, 0.005 g/L FeSO_4_·7H_2_O, 0.5 g/L MgSO_4_·7H_2_O, 0.45 g/L CaCl_2_·2H_2_O, 0.05 g/L bile and 0.4 g/L cysteine-HCl, plus 1.5 mL of the previously described vitamin mixture). Of this fecal-dialysate mixture, 60 mL were introduced in the model under anaerobic conditions.

### 2.4. Test Design

Inoculation with the fecal pool was carried out at −20 h (20 h prior to the start of the interventions), the feeding was started with SIEM, and the adaptation period was started for 18 h. Samples were taken from the lumen at −20, −18 and −16 and all samples were stored at −80 °C until analysis. After the adaptation period, the feeding was stopped for two hours (=starvation period) to allow fermentation of the remaining carbohydrates in SIEM by the microbiota. Next, the intervention period started, which lasted 72 h. Samples were taken at 0, 24, 48 and 72 h from the lumen and the dialysate. Feeding was started at 0 h and the different interventions were carried out: SIEM, 10× CHO, 0.1× CHO, Glucose (Table 1). The experiments were performed with four replicates for the dietary interventions. Since the feeding in the adaptation period was the same for all interventions, samples in the adaptation period were taken for only two of the replicates (leading to N = 8).

### 2.5. Gut-Mycobiota Composition

To determine the fungal composition in the lumen samples, DNA was isolated using the QIAamp Fast DNA stool mini kit from Qiagen (Venlo, The Netherlands), with a beadbeating step, according to the protocol described previously [22], which is suitable for optimal isolation of fungal DNA. The starting material was 400 µL lumen sample and the final elution was carried out in 50 µL elution buffer, to ensure high yield. DNA concentration was measured using the Qubit dsDNA HS Assay and a Qubit 3.0 Fluorometer (Invitrogen, Waltham, MA, USA).

The isolated DNA was used for sequencing the internal-transcribed-spacer unit 2 (ITS2), for determination of the fungal-community composition. For the library preparation of the ITS2 sequencing, the Fungal Metagenomic Sequencing Demonstrated protocol of Illumina was used, with some modifications (Nextera XT DNA Library Preparation Kit, Nextera XT Index Kit v2 Set A, Illumina, Eindhoven, The Netherlands). Input DNA in PCRI was increased to 50 ng/µL, and the PCRI program was adjusted to 30 cycles. Primers used were ITS F (5′-GCATCGATGAAGAACGCAGC-3′) and ITS R (5′-TCCTCCGCTTATTGATATGC-3′) [23]. After PCRII, all samples were checked with the bioanalyzer using the DNA1000 kit (Agilent, Santa Clara, CA, USA) to check the fragment sizes, which are variable for ITS2 sequences. The libraries were quantified, normalized and pooled equimolarly before loading on the Illumina Miseq system (Miseq reagent kit v3, Illumina). The Local Run Manager Generate FastQ Analysis Module v3 was used to generate fastq files, and further analysis was carried out with the Quantitative Insights Into Microbial Ecology 2 (QIIME2) software package (version 2019.7) [24,25]. The QIIME2 plugin Q2-ITSxpress was used to trim ITS sequences [26]. After this, the dada2 plugin was used for demultiplexing, quality filtering and denoising [27]. Classification of the ITS2 sequencing data was carried out with the use of the reference database UNITE (version 02-02-19).

### 2.6. Gut-Microbiota Composition

DNA isolated as above was also used to determine the gut bacterial composition, by sequencing the V3-V4 region of the 16S rRNA gene. The library preparation was carried out according to the Illumina protocol (Nextera XT DNA Library Preparation Kit, Nextera XT Index Kit v2 Set A, Illumina). The primer set used was 341F (5′-CCTACGGGNGGCWGCAG-3′) and 785R (5′-GACTACHVGGGTATCTAATCC-3′). Sequencing was carried out on the Miseq system from Illumina, using the Miseq reagent kit v3. With the use of the Local Run Manager Generate FastQ module v3, fastq files were created. Bioinformatic analyses were carried out with QIIME2 (version 2019.7). Taxonomic classification was carried out with the SILVA reference database (version 132).

### 2.7. Statistical Analysis

Alpha-diversity indexes analyses were calculated in QIIME2. Further statistical analyses were performed in R (version 4.0.4) using RStudio (version 1.4.1106). Data were expressed as median, with range, and significance tested with the Wilcoxon rank-sum test. Kruskal–Wallis analyses were performed to find correlations between individual taxa and categorical parameters, and Dunn’s test was performed as post-hoc analysis. A *p*-value of <0.05 was considered significant. Data was visualized using ggplot2 (package version 3.3.5). Beta diversity was expressed as Bray–Curtis dissimilarities and Jaccard similarities. Differences between groups were tested using PERMANOVA.

## 3. Results and Discussion

### 3.1. Adaptation Period

The aim of this research was to use the sophisticated in vitro model (TIM-2) to mechanistically study the mycobiome. The in vitro model was inoculated with a fecal pool from healthy individuals, and after introduction into the model, 18 h were used as an adaptation period for the mycobiota to adapt to the new environment. The mycobiota were analyzed for diversity, and this was compared with the bacterial component of the microbiota in the same period, to assess the stability. The fungal composition of the lumen samples was determined in the first four hours of the adaptation period (t-20, t-18 and t-16) and at the start of the intervention period (t0). To examine the abundance and the evenness of the fungal microbiota, the alpha-diversity was determined over time. In Table 2, the observed features at ASV level and effective Shannon-diversity-index are shown for fungi and bacteria. From this table, it can be seen that the observed ASVs for fungi are low, with a median of 22.5. The median effective Shannon-diversity-index was 22.0. This low fungal-diversity compared to bacterial diversity is found more in studies on the mycobiome [28]. In comparison, the diversity for bacteria is much higher, with a median of 488 observed ASVs and an effective Shannon-diversity-index of 5973.

**Figure 1:**
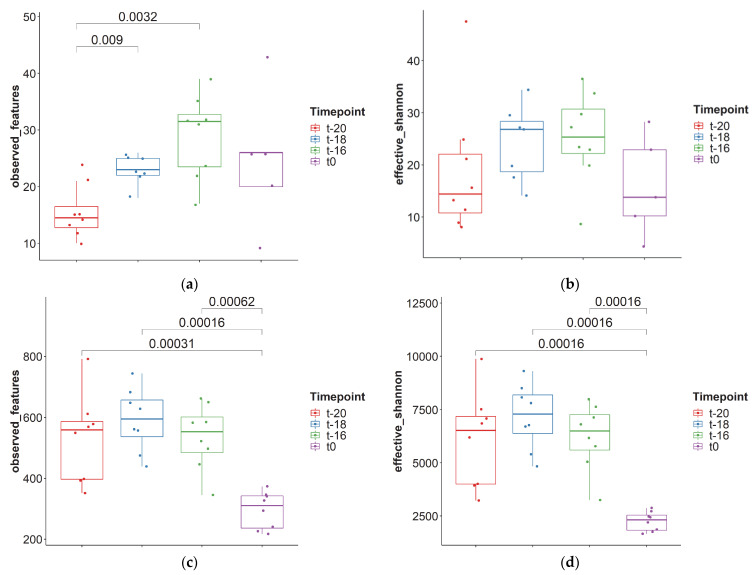
Alpha-diversity of the lumen samples during the adaptation period. (**a**) Observed features of fungi; (**b**) Shannon-diversity-index fungi; (**c**) observed features bacteria; (**d**) Shannon-diversity-index bacteria; significant pairwise comparisons are shown (Wilcoxon signed-rank test).

Figure 1A,B shows pairwise comparisons of the observed ASVs and the effective Shannon-diversity-index between the different time points in the adaptation period. The observed fungal ASVs changed significantly in the first two hours (*p* = 0.009) and between t-20 and t-16 (*p* = 0.0032), while other comparisons were not significantly different (*p* > 0.05). The effective Shannon-diversity-index of fungi did not change significantly. In addition, the alpha-diversity for bacteria was compared among the different time points, using pairwise comparisons (Figure 1C,D). Here, the changes were significant between t-20 and t0 (*p* = 0.00031), t-18 and 0 (*p* = 0.00016), and for t-16 and 0 (*p* = 0.00062) for the observed ASVs and for the effective Shannon-diversity-index between t-20 and t0 (*p* = 0.00016), t-18 and t0 (*p* = 0.00016) and t-16 and t0 (*p* = 0.00016). This is different compared to fungi, where most changes took place in the first hours of the adaptation period.

**Figure 2:**
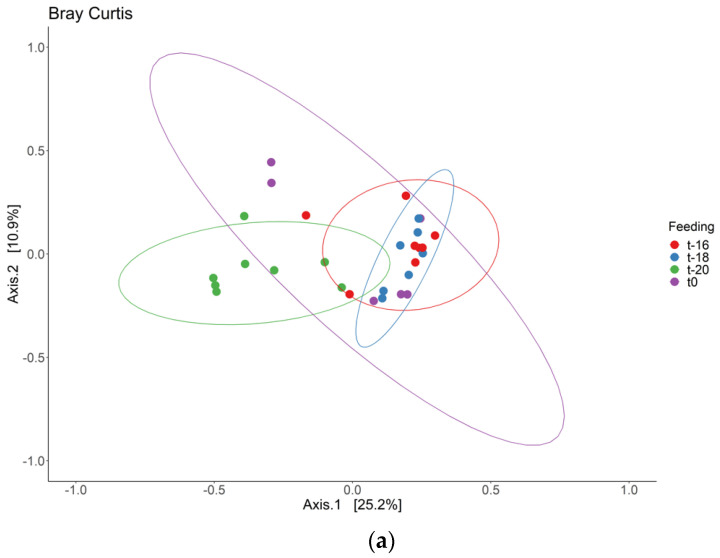

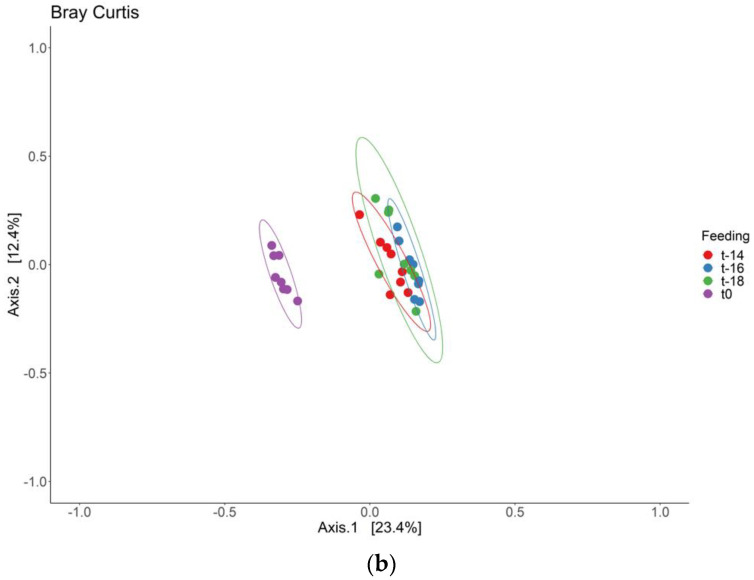
Beta-diversity during the adaption period visualized using PCoA plot with Bray–Curtis dissimilarity distances; (**a**) fungi; (**b**) bacteria. N = 8.

The beta-diversity was determined, to measure the variation between the different time points in the adaptation period. Figure 2A shows the beta-diversity (Bray–Curtis dissimilarity) for the different time points. The stability of the fungi in the model was shown to be affected primarily in the first two hours of the adaptation period, after which the beta-diversity remained stable, but a high variance between samples was seen at t0. For bacteria, the Bray–Curtis dissimilarity is also shown (Figure 2B). Here, the time points t-20, t-18 and t-16 cluster together, and the samples of t0 cluster together. The points at t0 show less variance, compared to the fungal samples. The greater variability of the mycobiome between samples could be explained by the low diversity as observed in the alpha-diversity plots, because a change in one of the dominant taxa in the samples drives great differences in beta-diversity. However, the beta-diversity expressed as Jaccard similarity (presence/absence of taxa) also shows differences in clustering of these time-points (Figure S1), which could mean that some taxa are also present in some samples, while not in others (see also below).

**Figure 3:**
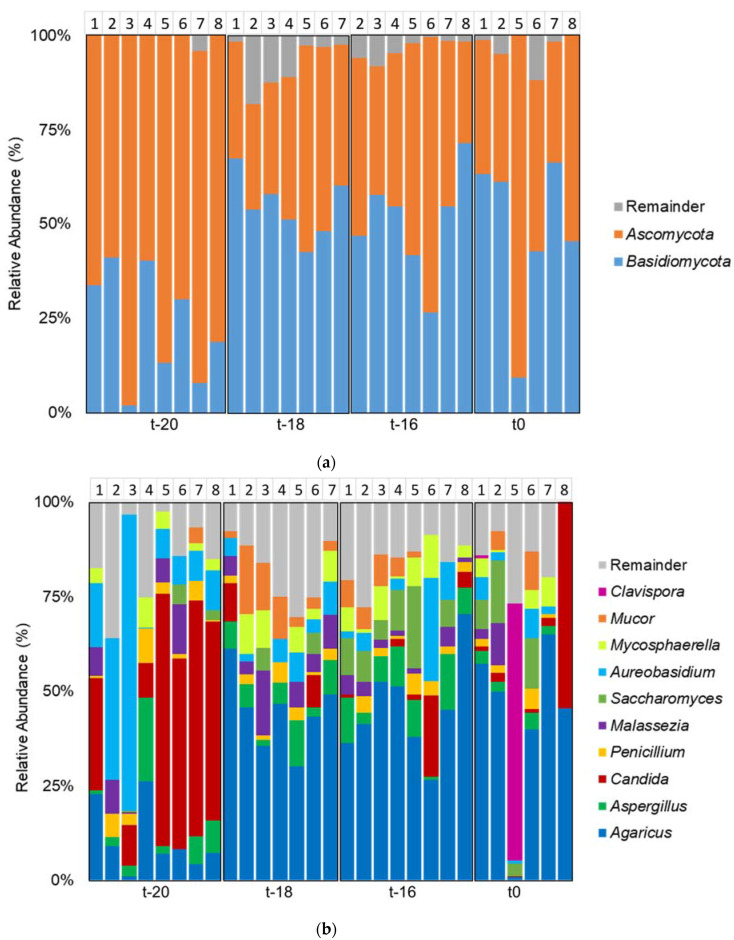
Relative abundance (%) of major groups of fungi during the adaptation period after inoculation in TIM-2; (**a**) phyla; (**b**) genera; N = 8 for t-20, t-18 and t-16, *n* = 6 for t0, due to two samples not passing the quality filtering.

**Figure 4:**
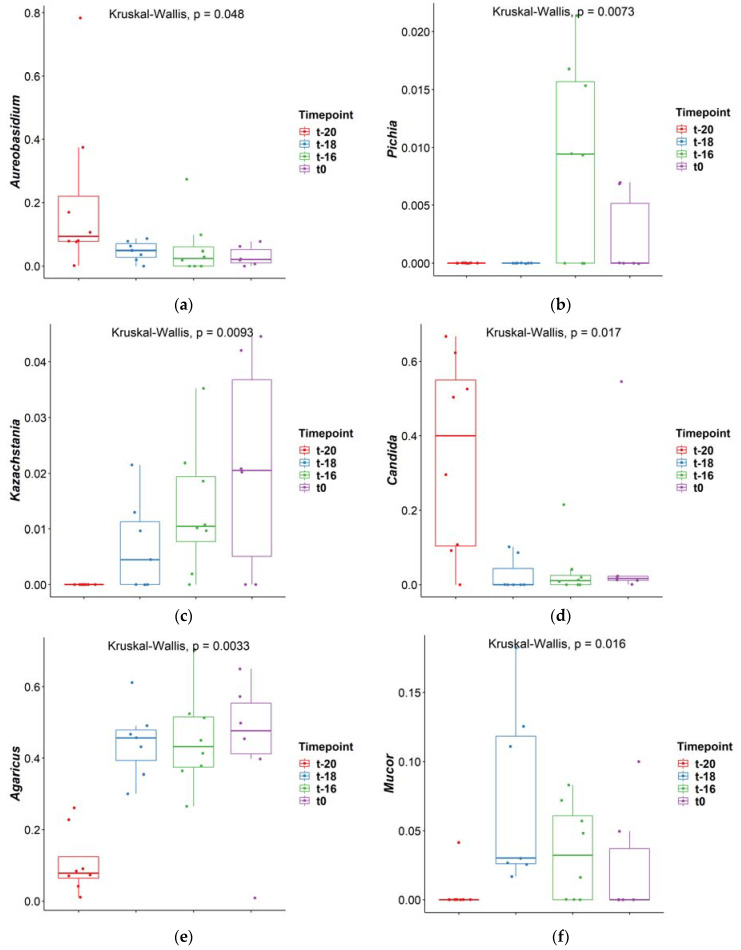
Box-plot of genera: (**a**) *Aureobasidium*; (**b**) *Pichia*; (**c**) *Kazachstania*; (**d**) *Candida*; (**e**) *Agaricus*; (**f**) *Mucor* that were significantly different (Kruskal–Wallis test) at different time points during the adaptation period (*p* < 0.05). Dunn’s post hoc test was performed to indicate the groups that were significantly different.

The fungal composition was further assessed by analysis of the taxonomic composition. The composition was evaluated at the phylum and genus level of taxonomical rankings. The phyla *Ascomycota* and *Basidiomycota* were dominant across all samples (Figure 3A). The dominance of these phyla in the human gut has also been previously described [29,30]. The ten most abundant fungal genera across the samples were *Agaricus*, *Aspergillus, Candida, Penicillum, Malassezia, Saccharomyces, Aureobasidium, Mycosphaerella, Mucor* and *Clavispora* (Figure 3B). These dominant genera made up most of the fungal composition in the different samples. Some genera showed a significant change during the adaptation period: *Aureobasidium*, *Pichia*, *Kazachstania*, *Candida*, *Agaricus* and *Mucor* (all *p* < 0.05; Kruskal–Wallis test; Figure 4). Dunn’s test was carried out as post-hoc analysis (Table 3). The genus *Candida* was highly abundant at the time point t-20, but during the course of the adaptation period it became less abundant in most samples. Although speculative at this point, the hypotheses might be entertained that (a) the inoculum contained non-viable Candida cells, which were detected by the molecular method at t-20, but were ‘recycled’ at t-18 and therefore no longer detected, or (b) the conditions of the proximal colon mimicked in the in vitro model (e.g., pH 5.8www) are non-permissive for Candida, which thrives better at the neutral pH found in the distal colon. The genus *Agaricus* became more dominant throughout the adaptation period. In a study on the gut mycobiome of the Human Microbiome Project healthy cohort the most abundant genera found in feces with ITS2 and 18S sequencing were *Saccharomyces, Mallasezia, Candida, Cyberlindnera, Penicillum, Cladosporium, Aspergillus, Debarymyces, Pichia, Clavispora* and *Galactomyces* [28]. The genera *Agaricus* has also been described before as present in the gut of vegetarians [31]. In these studies on the mycobiome, high inter-individual differences were observed in fecal samples. In the in vitro model used in this research, a fecal pool was used as inoculum for the experiment. The use of a fecal pool instead of individual samples should lead to less differences among samples [18]. However, some differences could be observed already at the start of the adaptation period (t-20), and these differences increased over the course of the experiment, leading to a greater variability at t0, as for instance was evidenced by one replicate showing a bloom of Clavispora (Figure 3B).

### 3.2. Intervention Period

For the intervention period, the alpha-diversity was determined for fungi and bacteria as well. Similar to the adaptation period, the observed ASVs were much lower for fungi, compared to bacteria (Table 4). In addition, the effective Shannon-diversity-index was lower for fungi, compared to bacteria.

**Figure 5:**
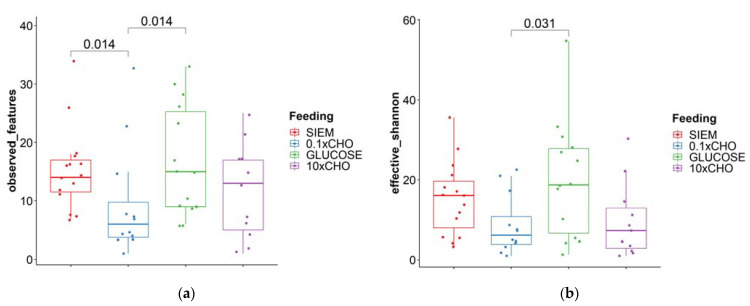

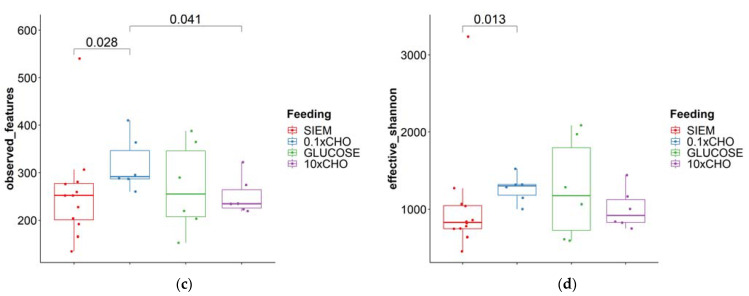
Alpha-diversity of the lumen samples during the intervention period; (**a**) observed features of fungi; (**b**) Shannon-diversity-index fungi; (**c**) observed features of bacteria; (**d**) Shannon-diversity-index bacteria. Pairwise comparisons Wilcoxon signed-rank test.

To determine whether the dietary interventions carried out in the intervention period had an influence on the diversity, pairwise comparisons were carried out between interventions; samples were included for the time points t24, t48 and t72. For fungi, the observed ASVs were significantly different between SIEM and 0.1 × CHO (*p* = 0.014) and 0.1 × CHO and glucose (*p* = 0.0014) (Figure 5A,B). The effective Shannon diversity index was significantly different between 0.1 × CHO and glucose (*p* = 0.038). The same pairwise comparisons were carried out for the bacterial community. Here, the observed OTUs were significantly different between SIEM and glucose (*p* = 0.0028) and between 0.1 × CHO and 10 × CHO (*p* = 0.041); the effective Shannon diversity index was different between SIEM and 0.1 × CHO (*p* = 0.013) (Figure 5C,D).

**Figure 6:**
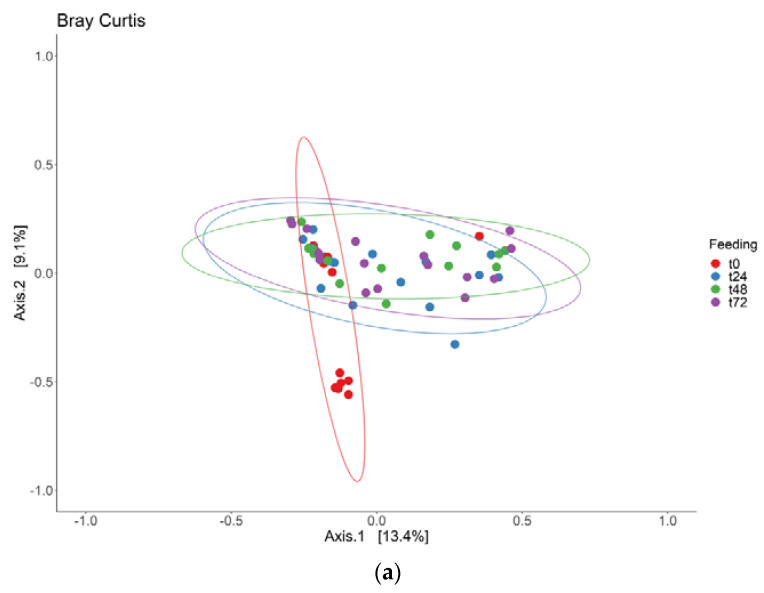

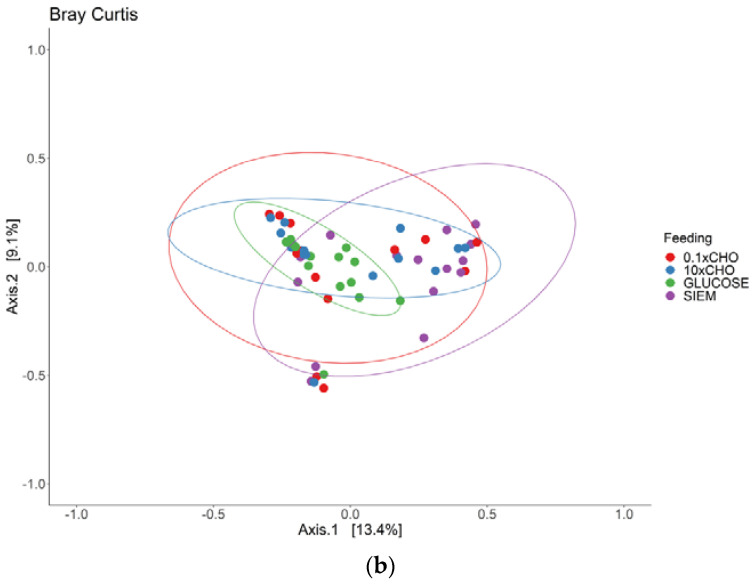
Beta-diversity of the fungal community during the intervention period, visualized using PCoA plot with Bray–Curtis dissimilarity distances; (**a**) between time points for all feeding interventions; (**b**) between feeding interventions for the time points t24, t48 and t72.

To check if the samples for the different time points and feeding strategies would cluster together, beta-diversity was determined with the use of Bray–Curtis dissimilarities (Figure 6) and the Jaccard index (Figure S2), as for the adaptation period. First, the samples were compared between the different time points (Figure 6A). Samples of t0 clustered together and were significantly different, compared to t24, t48 and t72 (*p* < 0.05). It should be noted though that the first two axes of the PCoA plot only explain 22.5% of the variance in the data. Additionally, the different feeding strategies of the samples were compared (Figure 6B). For this comparison, the samples of t0 were excluded, as the microbiota had not been exposed to the different intervention at that time point. The standard feeding SIEM clustered together with the 0.1 × CHO and the 10 × CHO. The samples where glucose was used as a carbon source also clustered together, and were significantly different from the other interventions (*p* < 0.05) (PERMANOVA).

**Figure 7:**
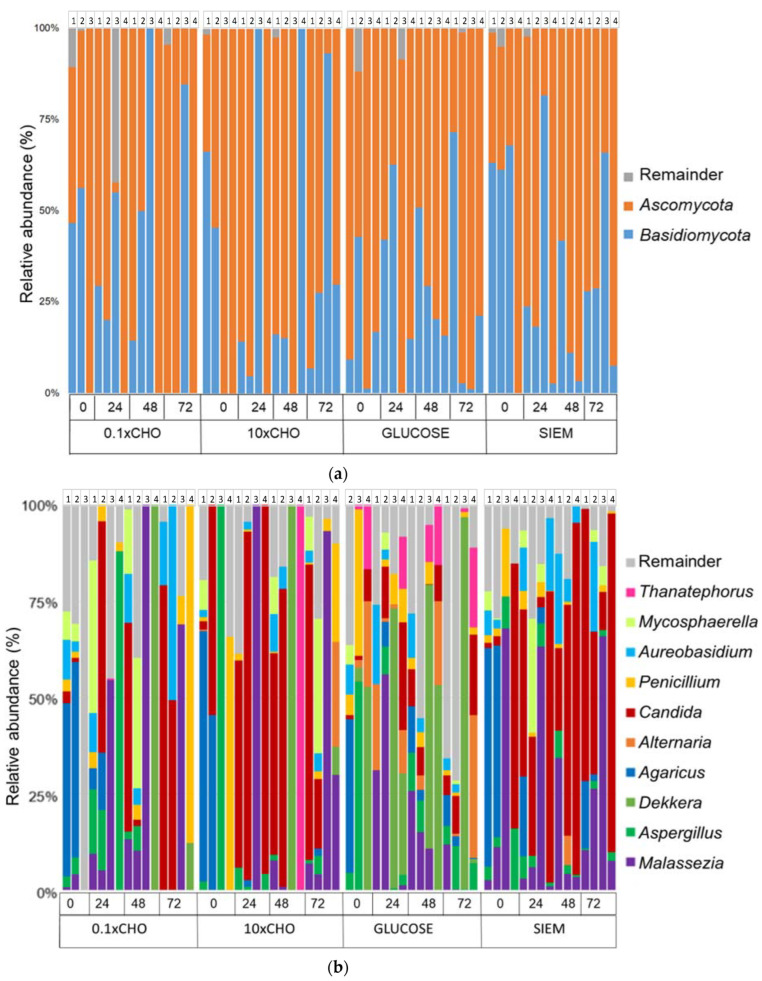
Relative abundance (%) of major groups of fungi during the intervention period after inoculation in TIM-2; (**a**) phyla; (**b**) genera.

**Figure 8:**
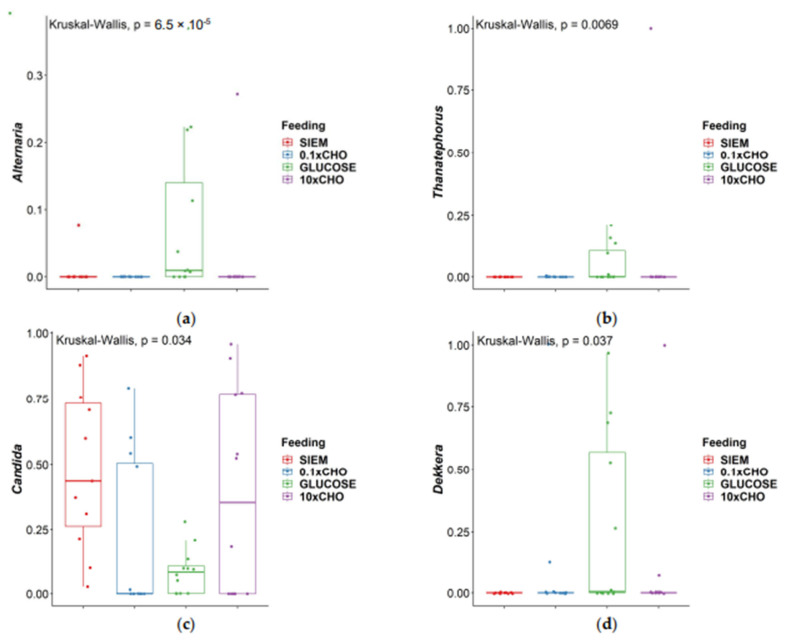
Box-plot of genera: (**a**) Alternaria; (**b**) Thanatephorus; (**c**) Candida; (**d**) Dekkera that were significantly different (Kruskal–Wallis test) for different dietary interventions (*p* < 0.05). Dunn’s post hoc test was carried out to indicate the groups that were significantly different.

In addition to the diversity analyses, the fungal composition in the samples of the intervention period was also analyzed. In Figure 7, the taxonomic classification at phylum and genus level for the different interventions over the course of the experiment are shown. The phyla *Ascomycota* and *Basidiomycota* are still dominant across all samples (Figure 7A). The ten most abundant genera are shown in Figure 7B; these are *Alternaria, Thanatephorus, Mycosphaerella, Aureobasidium, Penicillium, Aspergillus, Agaricus, Dekkera, Malassezia* and *Candida*. To test whether the genera were significantly different between dietary interventions, the Kruskal–Wallis test was carried out. The genera *Alternaria, Thanatephorus, Candida* and *Dekkera* were different between interventions (*p* < 0.05) (Figure 8). Dunn’s test was performed as post-hoc analysis (Table 5). In other studies on the mycobiome, *Candida* was positively correlated with carbohydrate consumption, mainly simple sugars [11,30]. This is different compared to what was observed in our research, where *Candida* was low in the intervention with glucose. Instead, the genera *Dekkera* and *Alternaria* were dominant in the samples with glucose, and probably prevented the increase in relative abundance of *Candida.* This shows that it is difficult to predict how diet can drive the mycobiome composition. Not all fungi found in the gut are stable gut-colonizers. Some fungi that are present in food are transient species, and will not settle in the complex microbial environment. In studies on the mycobiome, often samples are taken at one time point, which makes it difficult to discriminate between these transient species and gut-colonizers. In the in vitro model, the mycobiota are followed for 72 h, and the feeding is standardized, allowing for the control or elimination of fungi introduced by the diet. In addition, there are some challenges in sequencing the ITS compared to 16S for bacteria, including the variable length of the ITS, and lower levels of the fungal population in the total microbiota, which makes it more difficult to study fungi in this way. Lastly, it is unknown whether the nucleic acids that were detected at the start of the experiment may also come from non-viable fungi that are present in stool, but will be amplified after DNA isolation.

We set out to use the dynamic computer-controlled in vitro model of the colon (TIM-2) as a tool to study the fungal communities in the gut microbiota. This model has been extensively used to study the bacterial community, and has been validated for changes in bacterial composition and activity [11]. Validation for the fungal community is more difficult, due to the very high inter-individual variation in the fungal community among individuals [27]. For the bacterial community, our group has shown before that pooling microbiota can at least lead to the same starting microbiota [17], allowing better comparison between experiments and applied interventions. Considering that the initial fungal diversity varied between replicates, the data should be interpret with care. Nevertheless, we showed that the model also allowed modulation of the fungal population. As such, the model can be applied to mechanistically study what happens to the fungal community, e.g., upon antibiotic or fungicide treatment, and the interaction (e.g., co-occurrence) between fungi and bacteria, although it remains to be seen whether this occurs similarly in vivo. Using more sophisticated tools such as substrates labelled with the stable isotope 13C, even metabolic cross-feeding between fungi and bacteria or vice versa can be studied. We anticipate that those mechanistic studies help in contributing to the closure of the gap in knowledge on the role of fungi in gut microbiota and health and disease.

## 4. Conclusions

Although in vitro gut models have the limitation that they do not include host factors, they have been shown to be useful in the study of the microbiome. These studies on the microbiome are usually restricted to the bacterial component of the microbiome. However, in this study it was shown that fungi can also be modelled in a complex in vitro model of the colon (TIM-2). The modelling of the fungi has the additional challenge that the variation between samples is big and the cell-numbers are small, which likely leads to a less stable community as compared to bacteria. Future research should focus on optimizing the stability of the fungi in the in vitro model to increase the strength of results found. To see if the mycobiome could be modulated, dietary interventions were carried out. Only in the diet with glucose could significant differences be observed, and only for four genera. To further investigate the fungal population and how it is modulated, bacterial–fungal interactions should be investigated further.

## Figures and Tables

**Table 1:** Dietary interventions TIM-2 experiment.

Standard diet (SIEM)	SIEM [21]
High-carbohydrate diet (10× CHO)	SIEM with 10:1 CHO:TBCO [13]
High-protein diet (0.1× CHO)	SIEM with 1:10 CHO:TBCO [13]
Glucose as carbohydrate source (Glucose)	SIEM-CHO + Glucose

**Table 2:** Alpha-Diversity of fungal and bacterial communities in the adaptation period.

	Fungi		Bacteria	
	*Observed* *Features*	*Effective Shannon Diversity*	*Observed* *Features*	*Effective Shannon Diversity*
Median	22.5	22.0	510	5973
Minimum	9	4.34	217	1654
Maximum	43	47.5	791	9311

**Table 3:** Genera that are significantly different over time during the adaptation period. *p*-values of Dunn’s test for different genera; significant *p*-values underlined.

Comparison	*Aureobasidium*	*Pichia*	*Kazachstania*	*Candida*	*Agaricus*	*Mucor*
t-16–t-18	0.561	0.003	0.246	0.607	0.990	0.171
t-16–t-20	0.019	0.002	0.003	0.013	0.002	0.071
t-18–t-20	0.090	1.000	0.092	0.003	0.003	0.002
t-16–t0	0.792	0.100	0.904	0.377	0.993	0.445
t-18–t0	0.426	0.261	0.232	0.182	0.984	0.044
t-20–t0	0.015	0.247	0.004	0.155	0.004	0.364

**Table 4:** Alpha-Diversity of fungal and bacterial communities in the intervention period.

	Fungi		Bacteria	
	*Observed* *Features*	*Effective Shannon Diversity*	*Observed* *Features*	*Effective Shannon Diversity*
**Median**	13	10.8	260	1021
**Minimum**	1	1.0	135	453.6
**Maximum**	34	54.8	540	3236

**Table 5:** Genera that are significantly different over time during the intervention period.

Comparison	*Alternaria*	*Thanatephorus*	*Candida*	*Dekkera*
**0.1 × CHO–10 × CHO**	0.581	0.891	0.208	0.983
**0.1 × CHO–Glucose**	0.00003	0.007	0.686	0.060
**10 × CHO–Glucose**	0.0003	0.010	0.393	0.057
**0.1 × CHO–SIEM**	0.615	0.641	0.006	0.324
**10 × CHO–SIEM**	0.971	0.548	0.133	0.334
**Glucose–SIEM**	0.0004	0.002	0.019	0.005

^*^p-values of Dunn’s test for different genera; significant *p*-values underlined.

## Data Availability

The raw sequences and corresponding metadata will be archived in the Sequence Read Archive (SRA) repository at the NCBI upon acceptance of the manuscript: http://www.ncbi.nlm.nih.gov/bioproject/902713 and http://www.ncbi.nlm.nih.gov/bioproject/907310 (accessed on 1 December 2022).

## Supplementary Materials

The following supporting information can be downloaded at https://www.mdpi.com/article/10.3390/jof9010104/s1: Figure S1: Beta-diversity during the adaption period visualized using PCoA plot with Jaccard similarities; (a) fungi; (b) bacteria. N = 8; Figure S2: Beta-diversity of the fungal community during the intervention period visualized using PCoA plot with Jaccard similarities; (a) between time points for all feeding interventions; (b) between feeding interventions for the time points t24, t48 and t72.
