# Supplementary material for: Modelling the Gut Fungal-Community in TIM-2 with a Microbiota from Healthy Individuals
Source: J Fungi (Basel). 2023 Jan 12;9(1):104. doi: 10.3390/jof9010104 (PMC9866872; doi:10.3390/jof9010104)
Supplement: Supplementary file 1 [file jof-09-00104-s001.zip › jof-2108032-supplementary.pdf]

Supplemental material

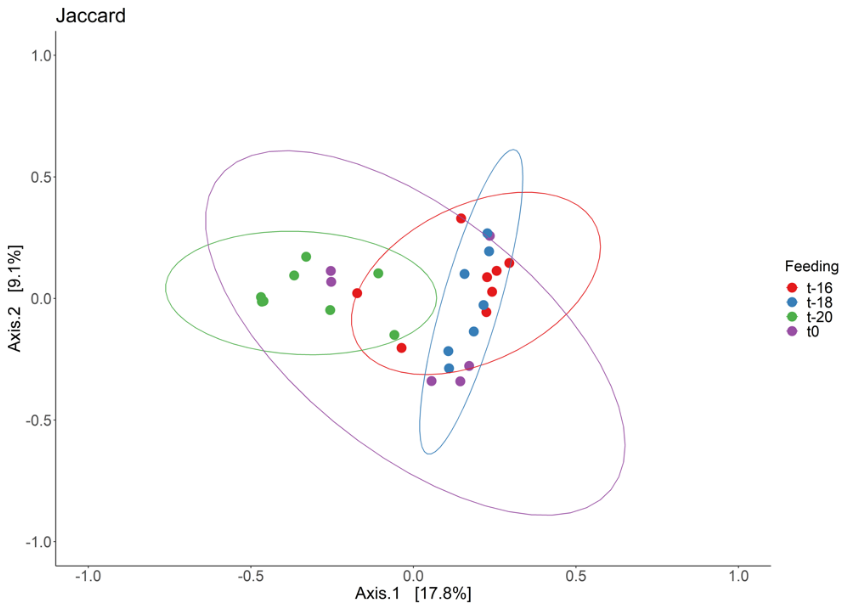

(**a**)

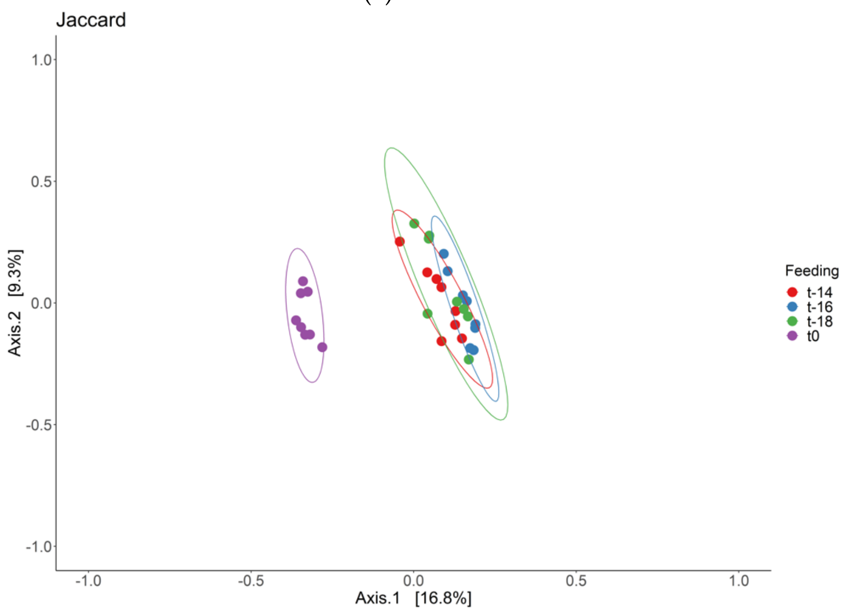

(**b**)

Figure S1: Beta-diversity during the adaption period visualized using PCoA plot with Jaccard.similarities**;** (**a**) Fungi; (**b**) Bacteria. N=8

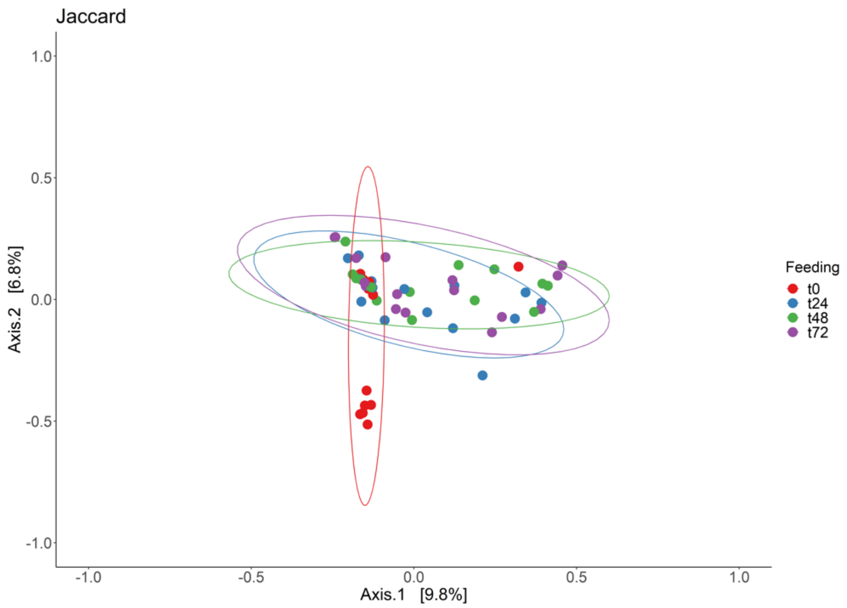

(**a**)

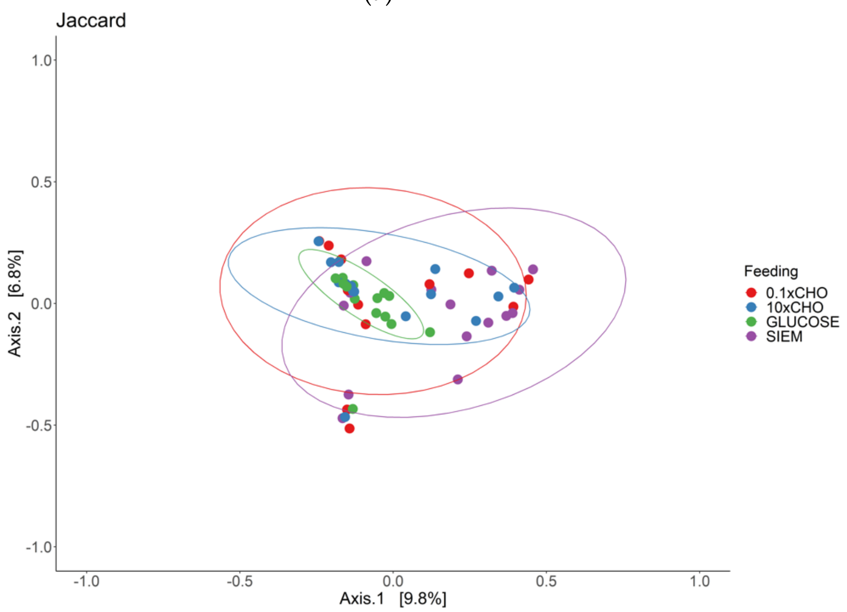

(**b**)

Figure S2: Beta-diversity of the fungal community during the intervention period visualized using PCoA plot with Jaccard similarities; (**a**) Between time points for all feeding interventions; (**b**) Between feeding interventions for the time points t24, t48 and t72.

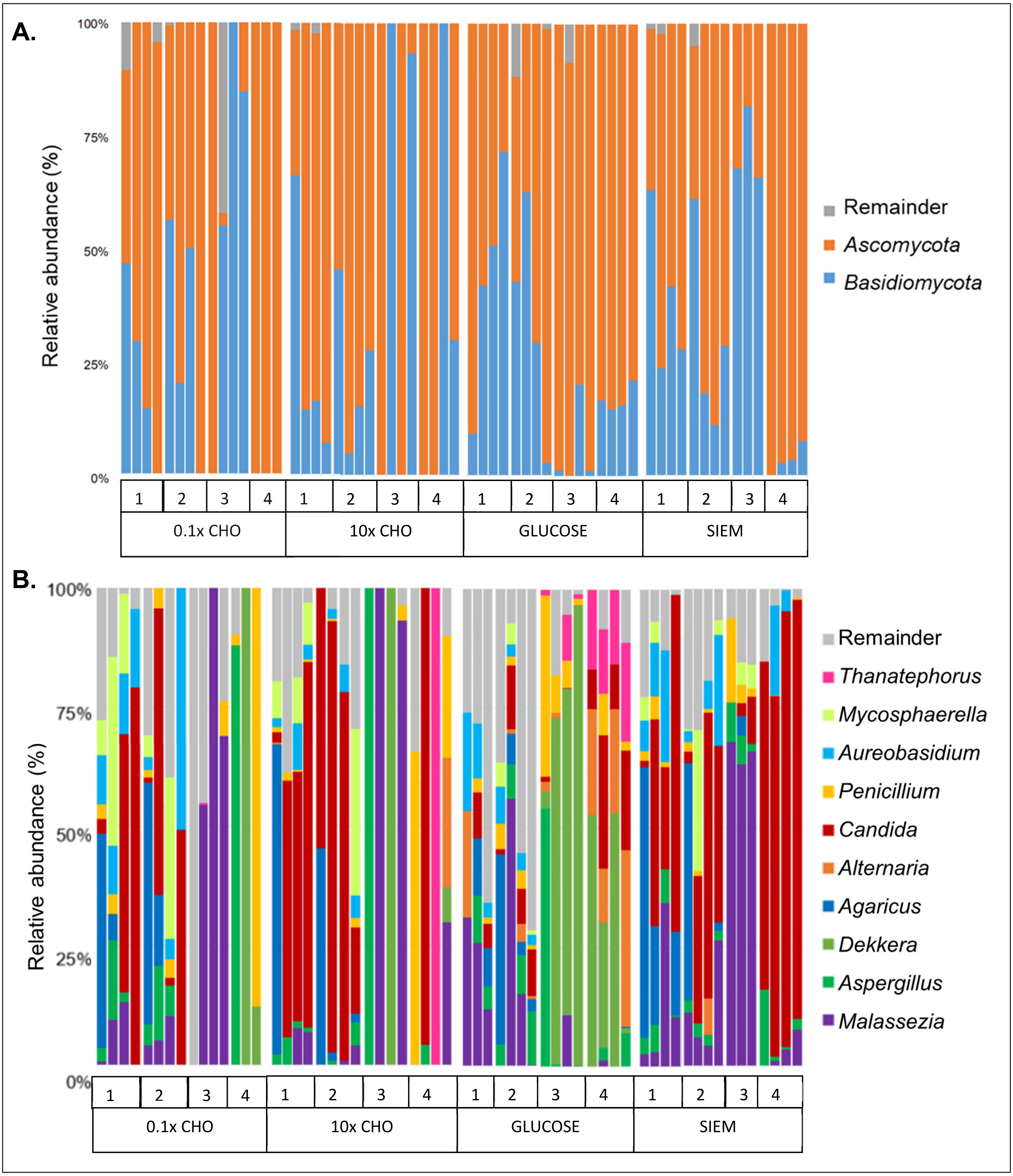

Figure S3: Relative abundance (%) of major groups of fungi during the test period after inoculation in TIM-2; (**a**) Phyla; (**b**) Genera; 1,2,3,4 stand for different replicates.

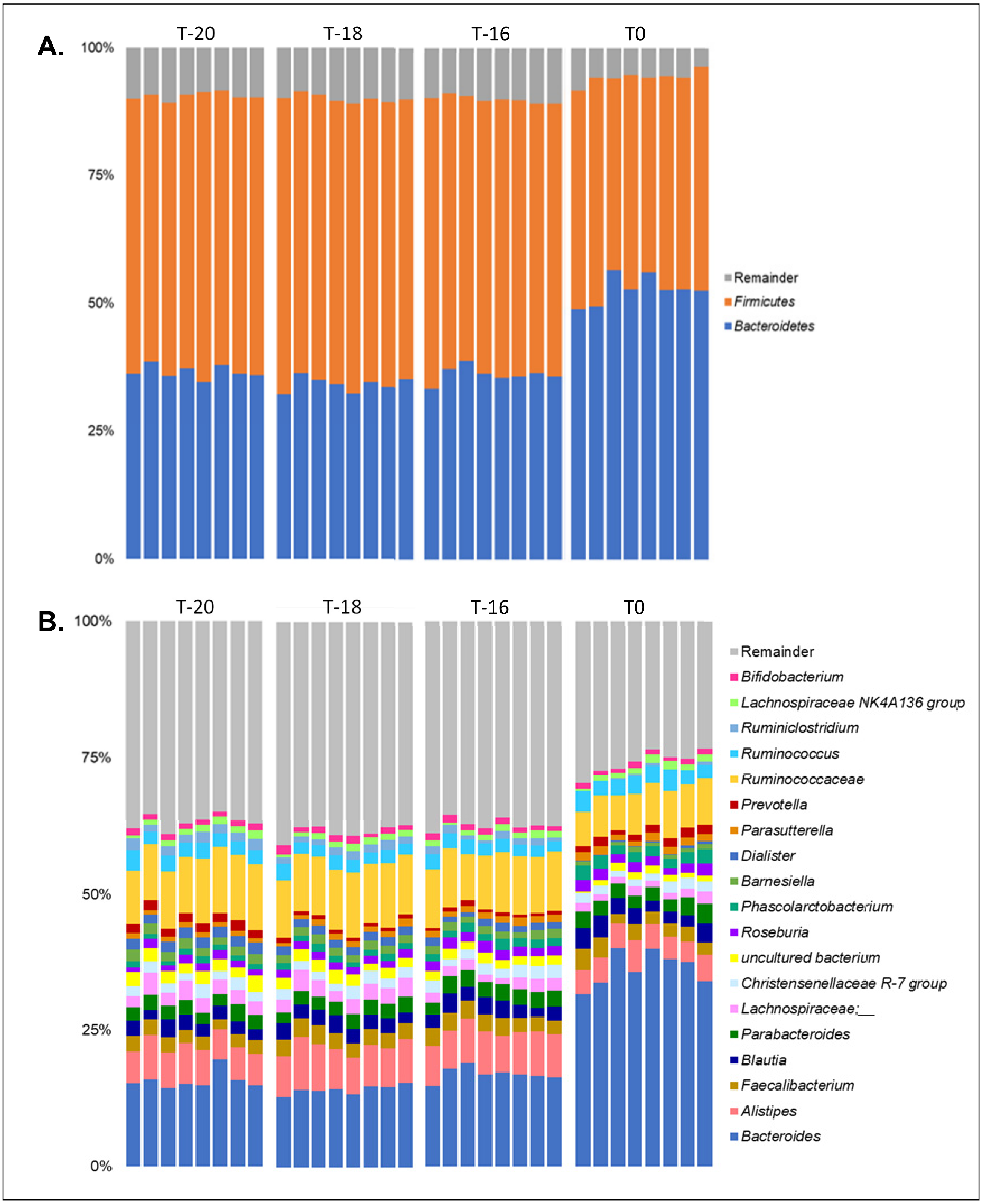

Figure S4: Relative abundance (%) of major groups of bacteria during the adaptation period after inoculation in TIM-2; (**a**) Phyla; (**b**) Genera.

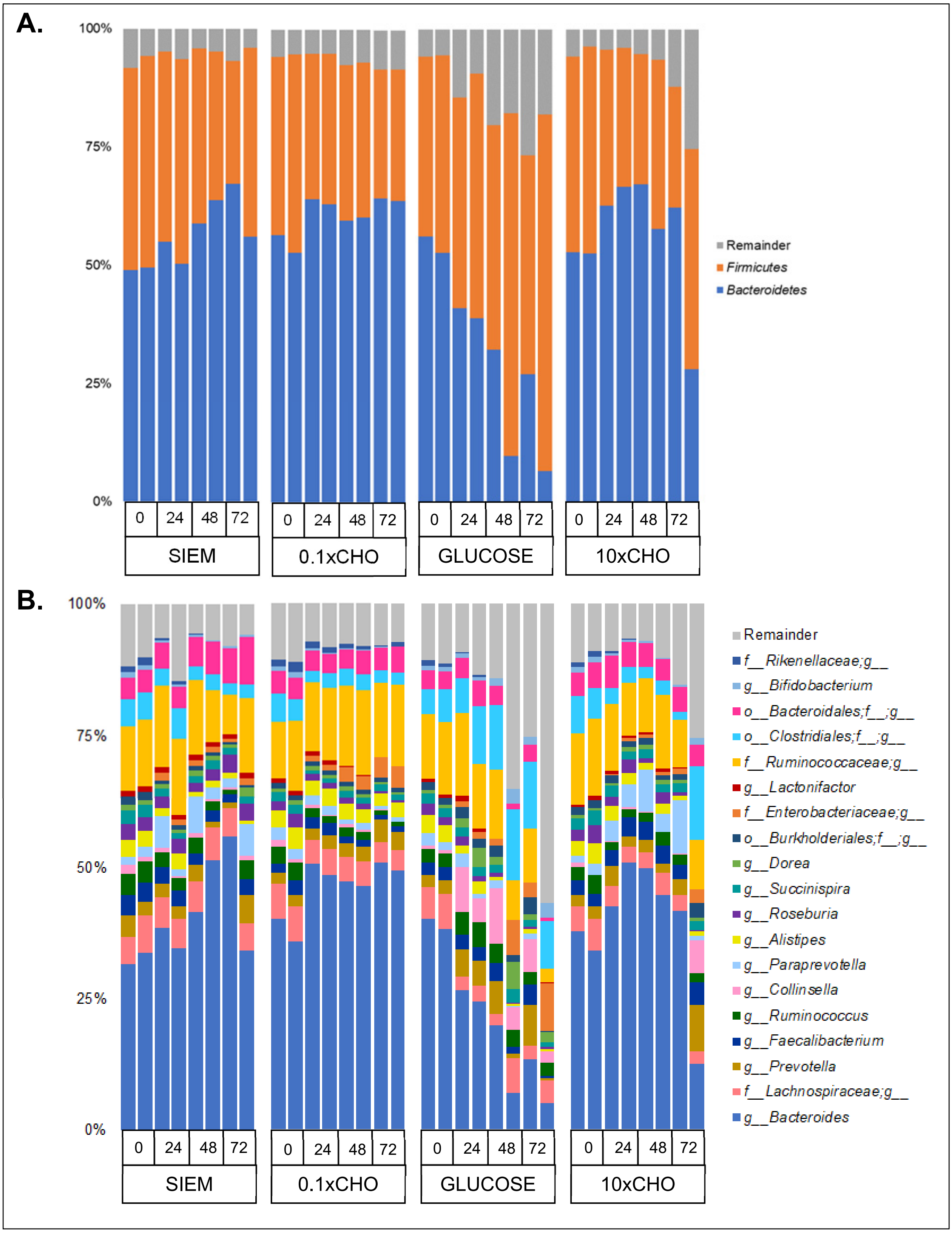

Figure S5: Relative abundance (%) of major groups of bacteria during the test period after inoculation in TIM-2; (**a**) Phyla; (**b**) Genera
